# Simultaneous Health Risk Behaviors in Adolescents Associated with Higher Economic Class in the Northeast of Brazil

**DOI:** 10.1155/2017/3587567

**Published:** 2017-07-26

**Authors:** Arley Santos Leão, Nara Michelle Moura Soares, Eliane Cristina de Andrade Gonçalves, Diego Augusto Santos Silva, Roberto Jerônimo dos Santos Silva, Sara Maria Thomazzi

**Affiliations:** ^1^Federal Institute of Alagoas, Maceió, AL, Brazil; ^2^Tiradentes University, Aracaju, SE, Brazil; ^3^Federal University of Santa Catarina, Florianópolis, SC, Brazil; ^4^Federal University of Sergipe, São Cristóvão, SE, Brazil

## Abstract

**Design:**

The social, cultural, and economic context can be an important variable in the perception and adoption of risk behaviors in adolescents.

**Objective:**

The purpose of this study was to identify the prevalence of simultaneous health risk behaviors and associated socioeconomic factors in adolescents living in the metropolitan region of Aracaju, State of Sergipe, Brazil.

**Methods:**

The sample consisted of 2,207 high school students aged 13–18 years. The risk behaviors measured were “low levels of physical activity,” “excessive daily TV time,” “high consumption of alcoholic beverages on a single occasion,” “involvement in fights,” “smoking cigarettes,” “carrying firearms,” and “marijuana consumption.” Information was obtained through self-administered questionnaire.

**Results:**

Considering the results, it was observed that female adolescents and those aged up to 16 years were less likely to have two or more health risk behaviors compared to males and those aged 17 years or more, respectively. It was also found that both high- and middle-income level adolescents had higher prevalence of having two or more health risk behaviors.

**Conclusions:**

It was concluded that male adolescents older than 16 years with better socioeconomic level were more exposed to the simultaneous presence of several health risk behaviors.

## 1. Introduction

Adolescence is characterized as a period when there is adoption of different behaviors that can negatively impact health. Some habits such as smoking, insufficient levels of physical activity, and poor eating habits are risk factors that favor the development of chronic diseases [1–3]. Other behaviors such as excessive consumption of alcohol, licit and illicit drugs, and carrying firearms are directly or indirectly related to the main external causes of death by violence or involvement in fights [4].

Large proportion of the young population is exposed to one or more health risk behaviors [5], which may extend to adulthood [6]. International [2, 7] and national [8–10] studies have shown that the individual presence or the simultaneous presence of health risk behaviors may contribute to morbidity (hospitalizations, serious injuries) and early mortality among young people.

This fact becomes even more relevant when considering that, in Brazil [8], 5.1% of adolescents aged 13–15 years have used tobacco in the last 30 days preceding the study, 26.1% consumed alcoholic beverages, and 2.5% have used marijuana. Behaviors related to sedentarism and physical inactivity were also analyzed, identifying that 78.0% of adolescents spend more than two hours per day watching television and 63.1% were physically inactive [8].

In addition to the risk factors for chronic diseases [1, 3, 7], there are early risk behaviors that affect young people such as unsafe sex, habit of driving after drinking alcohol, and engaging in fights [9, 11]. It is believed that the social, cultural, and economic context may be an important observation variable regarding the perception and adoption of health risk behavior among adolescents. According to the literature, the consumption of tobacco and physical inactivity have higher prevalence in young people, especially in those with lower incomes in southern Brazil [10].

Thus, it is necessary to identify not only health risk behaviors related to cardiovascular problems, but also those involving sociocultural and economic aspects, in order to favor objective actions aimed at solving problems arising from inadequate lifestyle.

Based on the above, this study aims to identify the prevalence of simultaneous health risk behaviors and associated sociodemographic factors among adolescents in the geoeconomic region of Aracaju, State of Sergipe, Brazil.

## 2. Materials and Methods

This study was characterized as cluster sampling survey [12], carried out with students enrolled in high schools of the State Education Network of Sergipe, geoeconomic region of Aracaju, which comprises the municipalities of Aracaju, Nossa Senhora do Socorro, São Cristóvão, and Barra dos Coqueiros. This population consisted of 13,373 students, which accounted for 60% of all students enrolled in this type of education in the State of Sergipe.

Sample size was calculated adopting sampling error of 3.0 (three) percentage points, outcome prevalence of 50%, and design effect of 2.0. In addition, 20% were added to the estimated amount to compensate for possible losses and refusals [13]. Based on this procedure, the minimum amount of students who should be investigated was estimated in 1,807. The sampling distribution respected the proportionality identified of enrollment per municipality.

The sample was selected by conglomerate in two stages. In the first stage, teaching units that offered high school with enrollment greater than 350 students were selected, totaling 16 schools. In the second stage, the total number of students enrolled in each school was taken into account, which was used to calculate the average number of students in each classroom, respecting the representative proportionalities of each school. Therefore, an average of 32.62 students/class was considered. The grades and shifts of participants were selected by simple random sampling, totaling 62 classes.

The sample power was calculated after data collection. For association tests, considering estimated prevalence of the outcome of 50%, power of 80%, and confidence level of 95%, this sample size would allow detecting as statistically significant a prevalence ratio from 1.21 as a risk factor and up to 0.80 as protection factor, considering a prevalence of exposure of 15%, prevalence of the outcome among unexposed subjects of 46.5%, and prevalence of the outcome among exposed subjects of 56.3%.

Inclusion criteria were (a) being regularly enrolled in high schools of the educational units chosen for the study; (b) having minimum age of 13 and maximum of 18 years; (c) having the Informed Consent Form (ICF) signed by parent or guardian; and (d) being present in the classroom on the day of data collection.

All data collection was carried out over a period of three months in 2011 by a team trained by professors from the Federal University of Sergipe and consists of ten evaluators who applied the questionnaire and clarified any doubts of students when needed.

Each teaching unit was visited on two days. On the first day, the objectives of the work were explained to clarify doubts and the ICF was delivered to students so that parents or guardians could sign it, authorizing their participation in the survey.

On the second day, in every class visited the day before, the team of evaluators collected the ICF signed and handed the instrument so that it could be filled. These evaluators remained in classroom for possible clarification related to the instrument throughout its application.

The instrument used for collecting information was drawn from the collection of parts of other instruments already tested and used in studies with adolescents [14–16]. The questionnaires were applied without the identification of subjects.  Table 1 showed the variables analyzed in this study, their measurements, and categorizations.

This study was approved by the Ethics Research Committee of the Federal University of Sergipe (CEP/UFS) under CAAE number 5724.0.000.107-10.

The prevalence of multiple health risk behaviors was estimated from the sum of individual behaviors, generating a score that was “0” (no behavior) to “8” (all health risk behaviors considered). It is noteworthy that all subjects considered in the study responded to the entire instrument.

To analyze the association between simultaneous exposure to different health risk behaviors and sociodemographic variables, Poisson regression with robust covariance estimator was performed. The models considered were (i) exposure to “one behavior or more behaviors” versus “no behavior”; (ii) “up to one behavior” versus “two or more behaviors”; and (iii) “up to two behaviors” versus “three or more health risk behaviors.”

From the crude and adjusted Poisson regression, prevalence ratio was estimated, with confidence intervals of 95% (CI95%). Only variables with *p* ≤ 0.20 in the crude analysis were considered for the adjusted analysis [17]. To be associated with the outcome, significance level of 5% was considered. The quality of all adjusted models was verified by the Omnibus test, which is a Chi-square likelihood ratio test that tests the hypothesis of similarity among models. Covariates were controlled in the analysis adjusted for sex, age, and economic status.

## 3. Results

Overall, 2,434 questionnaires were applied, of which 227 were considered as losses (143 responded by subjects older than 18 years and 84 excluded by limitations of completing and lack of essential information such as sex of adolescents). Thus, 2,207 questionnaires (90.67%) were eligible for effective data analysis. Since this study considers different outcome variables (health-related behaviors), it was chosen to analyze data from adolescents who responded to all outcomes, which resulted in 2,030 subjects (response rate = 83.40%).

Table 2 shows the sample distribution according to sex, age, economic status, and health risk behaviors. The majority of the sample was composed of females aged up to 16 years of intermediate economic level. The prevalence of little physically active individuals was 89.4%, sedentary behavior (TV time) was 69.4%, and smoking was 6.4%. Among students, 24.3% reported eating five or more alcoholic drinks in a single occasion, 3.4% reported carrying firearms, 16.2% reported some involvement in fights, and 2.3% reported marijuana use.

**Figure 1:**
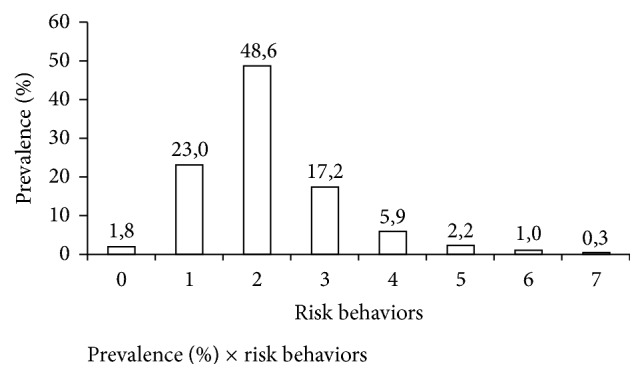
Prevalence of risk behaviors in adolescents in the geoeconomic region of Aracaju, State of Sergipe, 2011.

The prevalence of health risk behavior in adolescents investigated is shown in Figure 1. Only 1.8%  (*n* = 36) of adolescents did not present any health risk behavior. Most adolescents (*n* = 986) had two simultaneous health risk behaviors. More than 9.0% of adolescents had four or more simultaneous health risk behaviors.

The first analysis model (Table 3) was the comparison between groups of students with “one or more health risk behaviors” versus “no risk behavior.” Crude and adjusted analyses indicated that high and middle socioeconomic level adolescents had higher prevalence of having one or more health risk behaviors than those with no health risk behavior.

In the second analysis model (comparison among students with “two or more health risk behaviors” versus “one health risk behavior”), it was found in the crude and adjusted analysis that female adolescents and those aged up to 16 were less likely of having two or more health risk behaviors compared to males and those aged 17 years or more, respectively. Also in this analysis model, it was verified that in both crude and adjusted analysis high and middle socioeconomic level adolescents had higher prevalence of having two or more health risk behaviors (Table 3).

In the third analysis model (three or more health risk behaviors versus two or fewer health risk behaviors), it was found in the crude and adjusted analysis that female adolescents and those aged up to 16 years were less likely to have three or more health risk behaviors. Also in this model, it was found that, in both the crude and adjusted analysis, high and middle economic status adolescents had higher prevalence of having three or more health risk behaviors (Table 3).

## 4. Discussion

The present results indicate that boys were more exposed to health risk behaviors as found in other studies [2, 8, 11, 18, 19]. The study conducted by the US Disease Control Center (CDC) found that high school male students were more likely to have unintentional injuries, violence, tobacco use, excessive use of alcohol, and other drugs, as well as risky sexual behaviors [20], while the present study showed that male adolescents had higher prevalence in carrying firearms, violence, consumption of alcoholic beverages, and cigarettes.

The work of Hagger-Johnson et al. [3] contrasts, at least in part, the results found here when suggesting that students with lower economic levels were more likely to have a single risk behavior (smoking), concluding that, in economically disadvantaged subjects, this behavior is more prevalent and incident. It is believed that this is because young people of high socioeconomic level have easier access to consumer goods when compared to those of lower socioeconomic level, thus favoring the increased consumption of marijuana and alcohol and weapon possession.

The most important finding of this study relates to the question that the use of cigarettes and consumption of alcoholic beverages are related to older youth and the highest social class. This study partially confirms the findings of some studies [3, 9, 21–23], which found that smoking and drinking alcohol are prevalent behaviors in older. It is believed that this is because these behaviors are more influenced by cultural aspects.

Some authors reported simultaneous alcohol use and cigarette smoking in adolescents [19, 24, 25], suggesting that these behaviors, in addition to more prevalent, should have a greater focus on intervention and guidance programs aimed at young people. It is believed that this occurs because it is linked to risk factors for cardiovascular diseases, but this study indicates the importance of other health risk behaviors that are responsible for morbidity and mortality among young people. Furthermore, it is estimated that there is a positive relationship between alcohol and tobacco, so that the amount of tobacco consumed is related to alcohol consumption, suggesting that alcohol stimulates tobacco use and diverts the user's attention in relation to its consumption.

With regard to the associations found in this study, it is noteworthy that older and male adolescents were significantly associated with the simultaneity of two or more risk behaviors.

In a study conducted in the city of Florianópolis, SC, Brazil, male and older adolescents were associated with the simultaneity of three or more health risk behaviors [10], indicating that culturally, male adolescents adopt a behavior similar to that seen in this study, which strengthens the idea of similarity between cultural aspects associated with the presence/absence of certain behaviors in the same country.

In the present study, adolescents of high socioeconomic level had higher susceptibility of having simultaneous health risk behaviors. Different results were found in the study on simultaneous health risk behaviors developed by Dumith et al. [26] in the city of Pelotas, Brazil.

According to the literature, based on ecological social theories, the environment is responsible for behavior change; that is, low-income adolescents have less social support from friends, low education, and poor access to healthy food, which reflects the adoption of inappropriate health behaviors [23]. However, it seems that, in middle-income countries such as Brazil, this justification is not completely true for adolescents, which could be directly related to the cultural aspects and the instrument used for collecting information.

Corsi et al. [18] observed that the increase in educational and occupational levels was inversely associated with smoking, concluding that socioeconomic inequalities influence this behavior in adolescents. Liu et al. [27] analyzed the relationship between socioeconomic status and alcohol use in adolescents in China and Finland and found that there were significant differences between socioeconomic inequalities and alcohol consumption; the group with higher socioeconomic status was more likely to report the monthly use of alcohol, suggesting that interventions on alcohol use among adolescents should be aimed at those belonging to higher socioeconomic status.

In a survey conducted by Farias Júnior et al. [9], it was found that approximately one-third (36.5%) of the adolescents were classified as insufficiently active. These results are better than those found in this study. Regarding the consumption of illicit drugs, it was found that about 10% of adolescents reported having used some type [9], while in the current survey this percentage was below 5%.

This study showed the high prevalence of risk behavior in adolescents, according to findings of other studies [8, 27–31] and highlighting the urgent need for effective public policies in the school environment and out of school aimed at male, older, and higher socioeconomic level adolescent in order to reduce risk behavior among these people. These strategic actions will favor, in the medium term, the reduction of the prevalence of simultaneous risk behaviors in adolescents and reduce the likelihood of increasing prevalence in adulthood [9]. In addition, combating the adoption of risk behaviors will help to design and evaluate interventions and implement effective local programs and encourage educational policies for clarification of the population and to promote citizenship.

## 5. Conclusion

The results of this study allow concluding that male adolescents, those aged 16 years and with higher socioeconomic level, were more exposed to the simultaneous presence of various health risk behaviors in the region of Aracaju, State of Sergipe, Brazil.

## Figures and Tables

**Table 1:** Definition of the variables used in the study.

Variables	Questions used	Rating
*Sociodemographic variables*		
Sex^*∗*^	What is your sex?	Male
		Female
Age^*∗*^	How old are you?	≤16 years
		≥17 years
Socioeconomic level^*∗*^	Characterized based on ABEP^†^ criteria	High (“A1,” “A2,” “B1,” and “B2”)
		Medium (“C1” and “C2”)
		Low (“D” and “E”)

*Risk behaviors*		
Weapons possession^*∗∗*^	Over the past 30 days, on how many days did you carry a weapon, like a knife, gun, or nightstick?^††^	Never
		One or more times
Involvement in fights^*∗∗*^	Over the past 12 MONTHS, how often did you get involved in a close fight?^††^	Never
		One or more times
Consumption of more than five doses of alcohol at a single session^*∗∗*^	Over the past 30 days, on how many days did you take five or more doses of alcoholic in a single occasion?^††^	Never
		One or more times
Cigarette consumption^*∗∗*^	Over the past 30 days, on how many days did you smoke cigarettes?^††^	Never
		One or more times
Marijuana use^*∗∗*^	During your life, how many times did you smoke marijuana?^††^	Never
		One or more times
Level of physical activity^*∗∗*^	Characterized from the PAQ-A score^‡^, referring to the last seven days	Active (scores higher than three)
		Low levels of physical activity (scores less than three)
Hours watching TV/day^*∗∗*^	On average how many hours a day do you watch TV?^‡^	Up to two hours
		More than two hours

^*∗*^Socioeconomic variables; ^*∗∗*^variables on risk behavior; ^†^“Associação Brasileira de Empresas de Pesquisa,” 2015 [14]; ^††^YRBSS, Youth Risk Behavior Surveillance System, Brazil [15]; ^‡^Physical Activity Questionnaire for Adolescents, PAQ-A [16].

**Table 2:** Distribution of the sample according to sex, the age, the economic level, and behaviors of risk to health. Geoeconomic region “Grande Aracaju,” 2011.

Variables	*n* (%)
Sex	
Female	1283 (63,2)
Male	747 (36,8)
Age (years)	
≤16	1165 (26,4)
≥17	865 (73,6)
Economic level	
High	485 (23,9)
Medium	1292 (63,6)
Low	253 (12,5)
Physical activity level	
Active	216 (10,6)
Underactive	1814 (89,4)
TV time (hours/day)	
≤2	622 (30,6)
>2	1408 (69,4)
Smoke	
No	1901 (93,6)
Yes	129 (6,4)
Drinking 5 or more drinks on one occasion	
No	1536 (75,7)
Yes	494 (24,3)
Carry weapons	
No	1961 (96,6)
Yes	69 (3,4)
Involvement in fight	
No	1701 (83,8)
Yes	329 (16,2)
Marijuana use	
No	1983 (97,7)
Yes	47 (2,3)

**Table 3:** Crude and adjusted prevalence ratio for the association between simultaneous health risk behaviors and sociodemographic variables in adolescents from the geoeconomic region of Aracaju, State of Sergipe, 2011.

Variables	1 RB or more versus no RB	≥2 RB versus 1 RB	≥3 RB versus ≤2 RB	1 RB or more versus no RB	≥2 RB versus 1 RB	≥3 RB versus ≤2 RB
	Crude PR (CI 95%)	Crude PR (CI 95%)	Crude PR (CI 95%)	Adjusted PR (CI 95%)	Adjusted PR (CI 95%)	Adjusted PR (CI 95%)
*Sex*						
Female	0.99 (0.95–1.03)	0.78 (0.72–0.86)	0.72 (0.62–0.82)	—	0.79 (0.73–0.87)	0.73 (0.63–0.84)
Male	1	1	1		1	1

*Age*						
≤16 years	1.00 (0.97–1.04)	0.87 (0.80–0.95)	0.75 (0.65–0.86)	—	0.87 (0.80–0.95)	0.75 (0.65–0.86)
≥17 years	1	1	1		1	1

*Socioeconomic level*						
High	1.10 (1.02–1.18)	1.46 (1.22–1.76)	1.48 (1.13–1.93)	1.10 (1.02–1.18)	1.43 (1.19–1.72)	1.43 (1.09–1.87)
Medium	1.08 (1.01–1.15)	1.26 (1.06–1.51)	1.21 (0.94–1.56)	1.08 (1.01–1.15)	1.26 (1.06–1.50)	1.21 (0.94–1.56)
Low	1	1	1	1	1	1

PR: prevalence ratio; CI 95%: 95% confidence interval; RB: health risk behaviors.

## Additional Points

Practical implications are as follows: (i) complete understanding of the influence of risk behavior in adolescents, from all social classes, and (ii) construction of specific public policies to this population.
